# Supplementary material for: Predicting visual function by interpreting a neuronal wiring diagram
Source: Nature. 2024 Oct 2;634(8032):113–23. doi: 10.1038/s41586-024-07953-5 (PMC11446822; doi:10.1038/s41586-024-07953-5)
Supplement: Supplementary file 6 — CRF and ERF predictions for individual TmY4 and TmY9 cells. Analogous to Supplementary Data 3, but for TmY target types. Shown are the top four monosynaptic pathways, the strongest pathway passing through each of the top ten intermediary types (ranking from Extended Data Fig. 7), and the trisynaptic pathway Tm1–TmY–Dm3–TmY (see the section entitled Prediction of spatial normalization). [file 41586_2024_7953_MOESM6_ESM.zip › DataS4/TmY9q/720575940614726342.pdf]

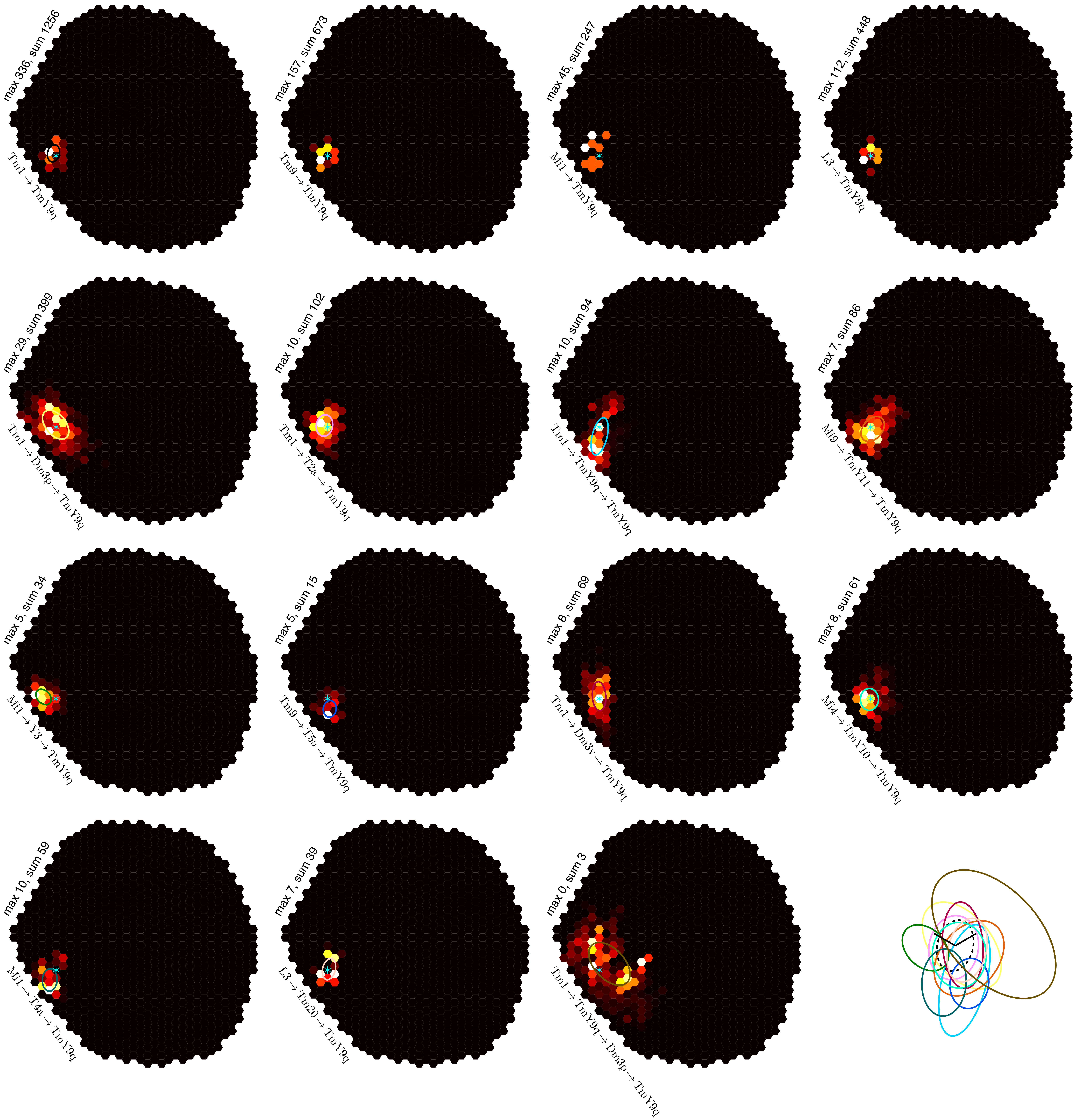
max 336, sum 1256
Tm1 → TmY9q
max 157, sum 673
Tm9 → TmY9q
max 45, sum 247
Mi1 → TmY9q
max 112, sum 448
L3 → TmY9q
max 29, sum 399
Tm1 → Dm3p → TmY9q
max 10, sum 102
Tm1 → T2a → TmY9q
max 10, sum 94
Tm1 → TmY9q → TmY9q
max 7, sum 86
Mi9 → TmY11 → TmY9q
max 5, sum 34
Mi1 → Y3 → TmY9q
max 5, sum 15
Tm9 → T5a → TmY9q
max 8, sum 69
Tm1 → Dm3v → TmY9q
max 8, sum 61
Mi4 → TmY10 → TmY9q
max 10, sum 59
Mi1 → T4a → TmY9q
max 7, sum 39
L3 → Tm20 → TmY9q
max 0, sum 3
Tm1 → TmY9q → Dm3p → TmY9q
